# Identifying the origin of Yemeni green coffee beans using near infrared spectroscopy: a promising tool for traceability and sustainability

**DOI:** 10.1038/s41598-024-64074-9

**Published:** 2024-06-10

**Authors:** Mariana Santos-Rivera, Christophe Montagnon, Faris Sheibani

**Affiliations:** 1 Smartspectra Limited, 52b Fitzroy Street, London, W1T 5BT UK; 2 RD2 Vision, 60 rue du Carignan, 34270 Valflaunes, France; 3 Qima Coffee, 21 Warren Street, Fitzrovia, London, W1T 5LT UK

**Keywords:** Arabica, Coffee authenticity, Chemometrics, Discrimination, Geographical origin, Green coffee beans, NIRS, PCA-LDA, Yemeni regions, Biochemistry, Chemistry, Infrared spectroscopy, Near-infrared spectroscopy

## Abstract

Yemeni smallholder coffee farmers face several challenges, including the ongoing civil conflict, limited rainfall levels for irrigation, and a lack of post-harvest processing infrastructure. Decades of political instability have affected the quality, accessibility, and reputation of Yemeni coffee beans. Despite these challenges, Yemeni coffee is highly valued for its unique flavor profile and is considered one of the most valuable coffees in the world. Due to its exclusive nature and perceived value, it is also a prime target for food fraud and adulteration. This is the first study to identify the potential of Near Infrared Spectroscopy and chemometrics—more specifically, the discriminant analysis (PCA-LDA)—as a promising, fast, and cost-effective tool for the traceability of Yemeni coffee and sustainability of the Yemeni coffee sector. The NIR spectral signatures of whole green coffee beans from Yemeni regions (n = 124; Al Mahwit, Dhamar, Ibb, Sa’dah, and Sana’a) and other origins (n = 97) were discriminated with accuracy, sensitivity, and specificity ≥ 98% using PCA-LDA models. These results show that the chemical composition of green coffee and other factors captured on the spectral signatures can influence the discrimination of the geographical origin, a crucial component of coffee valuation in the international markets.

## Introduction

Coffee geographical origin is an essential indicator of the quality, reputation, and exclusivity of coffee beans and can, therefore, demand significant premiums^1,2^. When grown in particular regions or nations, the “terroir” of the microclimate can result in distinctive attributes in the flavor and aroma of coffee. This “terroir” difference is a principal discriminator for coffee cultivated in different regions around the world^3,4^. The “terroir” plays an important role in determining the distinctiveness of green coffee beans, as it affects numerous variables that influence the quality of coffee, including altitude, soil type, temperature, shade coverage, and other climate factors^3,4^.

Yemen is considered to have played a crucial role in shaping the history of coffee since coffee cultivation and coffee trade originated in Yemen^5^. Yemeni coffee crops are predominantly grown in mountainous regions, typically by small-scale farmers using ancient farming techniques and manual post-harvest methods^6^. Moreover, the coffee lands hold unique genetic varieties not found elsewhere in the coffee-growing world^7,8^. Yemeni coffee is, therefore, highly sought after for its distinctive flavor profile, chemistry, and genetic uniqueness and is regarded as one of the most exclusive coffees available worldwide^9–16^. However, the country is also considered home to the worst humanitarian crisis in the world, due largely to the recent civil conflict, with 71–78% of Yemenis facing poverty. In such a challenging socioeconomic climate, Yemeni coffee cultivation and export represent an attractive economic opportunity for the estimated 150,000 smallholder coffee farmers of Yemen^17,18^.

Authenticity and traceability are essential for building a strong reputation in the coffee industry, where detailed information on the 'origin' of a coffee, including country of production, region, process, altitude, and genetic variety, among other attributes, is a fundamental component of the market value of the product^19^. In the absence of verification protocols, this 'origin' value creates an opportunity for coffee fraud and adulteration, in which sellers fraudulently miscategorized coffee to fetch a higher premium^20,21^. Food fraud is a global issue that goes beyond coffee and involves any situation in which food products are purposefully mislabeled or misrepresented for monetary advantage^22^. Yet, because of the high value of premium coffee and the intricacies of the supply chain, the coffee sector is particularly prone to food fraud^20,22^.

The Coffee Exporters Guide categorizes Yemeni coffee as a coffee with "Exemplary Quality"^23^. Still, decades of political upheaval have impacted the accessibility and reliability of Yemeni coffee beans. As such, demand for Yemeni coffee has collapsed in the international markets, with exports dramatically falling over the last several decades^24–26^. Almost 50% of prospective Yemeni coffee buyers cite 'lack of traceability' as the main area of improvement in the coffee industry of the country, and it is estimated that as much as 50% of coffees that are sold and exported from as 'Yemeni' are in fact African coffees that are imported into Yemen, mislabeled and re-exported as Yemeni origin coffee^26^. The extent of adulteration in Yemeni coffee has significantly impacted its reputation, with buyers facing the risk of purchasing high-priced fraudulent coffee^20^. This food fraud harms the reputation of producers, exporters, importers and erodes consumer trust and confidence. Moreover, it obscures the cultural and historical significance of coffee from this unique region^20,21^. If not addressed, coffee fraud in Yemen could lead to the devaluation and potential collapse of the Yemeni coffee industry. This would be a significant setback not only for Yemeni farmers, for whom coffee represents a heritage of over 600 years and one of the few remaining socioeconomic lifelines in a country plagued by conflict, but also for the genetic diversity of Arabica coffee. Yemen, known for being one of the most genetically diverse coffee-producing nations in the world, plays a crucial role in the preservation and development of this crop.

Currently, a few methods are used to identify the geographical origin of green coffee beans, including chemical analysis, such as isotope fingerprinting, Nuclear Magnetic Resonance (NMR) and, more recently, Near Infrared Spectroscopy (NIRS)^20,27–30^. These approaches can be used alone or in combination to determine the origin of green coffee samples and, by extension, to avoid fraud. The NIRS approach is considered a valuable technology for detecting food fraud in the coffee industry^20,22^. This vibrational spectroscopy technique can be used to evaluate the chemical composition of green coffee beans and identify specific chemical spectral signatures that indicate the geographic origin, variety, and quality of the beans^31–35^. The speed and non-destructive nature of NIRS are two of its most important advantages applicable to large-scale coffee testing. By comparing the chemical makeup of coffee samples to a reference database, NIRS can quickly and precisely determine their origin authenticity^36^. When combined with chemometrics, the procedure offers a rapid, robust, and dynamic technique to confirm the geographical origin of green coffee beans. Whilst studies assessing NIRS as a tool for green coffee origin verification have been previously undertaken, this is the first study focusing on Yemen as an origin^37–44^.

This study aims to identify and discriminate the NIR spectral signatures of whole green coffee beans from Yemen in comparison with other origins, creating the prospect of developing a practical, cost-effective tool for coffee traceability. The work also investigates the ability of NIRS to identify and discriminate intra-country origin differences, and to the authors' knowledge, it is the first study to do so with Yemeni coffee. A traceability tool backed by NIRS can empower buyers with assurance of the authenticity of their coffee origins and, as trust in Yemeni coffee is re-established, can support smallholder coffee farmers in receiving higher premiums for their produce.

## Materials and methods

### Green coffee samples

A total of 221 samples of whole green coffee beans of *Coffea arabica,* with verified geographical origin and farmer traceability data, were included in this study (Table 1, Supplementary Table S1 online). Specialty coffee samples (> 80 points in the Specialty Coffee Association of America (SCAA) scale) that were harvested in 2020 from Yemen (n = 124) were ethically sourced by Qima Coffee from smallholder coffee farmers in five regions of this country (Al Mahwit, Dhamar, Ibb, Sa'dah, and Sana'a) following the guidelines and regulations included in The Coffee Guide by the International Trade Centre and the International Coffee Organization ICC-102–9 Rules on Certificates of Origin. These samples were processed using the Natural post-harvest methods and included genetic varieties from the Typica Bourbon group (SL-28, SL-34, Kent) and the recently described New-Yemen group (Yemenia)^7,45^. In addition, specialty coffee samples from Africa, Asia, Central America, South America, and Oceania (n = 97) processed using Natural, Honey, and Washed post-harvest methods were acquired. They included a range of genetic varieties such as Caturra, Catimor, Catuai, Geisha, Heirloom, Java, Pacamara, Pink Bourbon, SL varieties, and Tabi. In preparation for the NIR spectra collection, 100 g of whole green coffee beans per sample with moisture between 10 and 12% were vacuum-packed in a thin barrier packing material and sent to France for spectra collection.

**Table 1:** Balanced databases distribution of NIR spectra (866–2532 nm) from whole green coffee beans per discrimination group.

GROUP	ORIGIN	Training	Cross-validation	Test	Total
1	YEMEN	282	70	144	496
	OTHER ORIGINS	282	70	36	388
	TOTAL	564	140	180	884
2	YEMEN	48	12	436	496
	AFRICA	48	12	40	100
	ASIA	48	12	68	128
	CENTRAL AMERICA	48	12	12	72
	SOUTH AMERICA	48	12	24	84
	TOTAL	240	60	580	880
3	AL MAHWIT	22	6	4	32
	DHAMAR	22	6	28	56
	IBB	22	6	108	136
	SA'DAH	22	6	8	36
	SANA'A	22	6	208	236
	TOTAL	110	30	356	496

Each Training and Cross-validation database has an equal number of spectra per geographical origin. Balanced databases or interactions were generated using stratified random sampling three times.

## NIR Spectral signature collection

The NIR spectra from whole green coffee beans (n = 884) were collected in 2021 by Bruker France SAS, using a Bruker Optics (Billerica, MA) Multi-Purpose Analyzer (MPA) II, Fourier Transform Near Infrared Spectrometer (FT-NIR) and OPUS® software package (Bruker Optics, Germany). Four spectral signatures per sample were collected at room temperature (20–22 °C) in diffuse reflectance mode without any sample preparation, using an integrating sphere and a sample rotator that assures high reproducibility for heterogeneous samples such as whole coffee beans. Each spectrum was created by averaging 128 scans in the wavelength range between 866 and 2532 nm (949 points of information = 2.7 nm resolution approx.). Instrumental internal reference standards were used to make background scanning.

## Chemometrics

Before the generation of the prediction models using chemometrics based Multivariate Analysis (MVA), the raw spectra (900–2400 nm) were extracted and inspected for outliers, then transformed using Unscrambler® X v.10.4 software (CAMO Analytics, Oslo, Norway) and the mathematical pre-treatments of Baseline Correction, Standard Normal Variate (SNV), and 2^nd^ derivative (polynomial order: 2, Savitzky-Golay smoothing points: 24). The Baseline Correction, was applied early in the process to ensure that subsequent pre-treatments are applied to a more uniform baseline, it removed baseline variations to enhance spectral features^46,47^. The SNV was used after the baseline to correct for scatter effects and intensity variations, improving the comparability of spectra^46,48^. By estimating the rate of change of absorbance, the second derivative was applied after SNV to emphasize small changes and enhance spectral features. Savitzky-Golay smoothing was used in this instance to improve the signal-to-noise ratio and reduce noise while keeping significant spectral features^48^. It is noteworthy that the sequence in which these pre-treatments are done might affect the outcome, and the choice is contingent upon the features of the dataset and the particular objectives of the study. Here, the authors employed cross-validation and testing techniques to evaluate the influence of distinct pre-treatments on model performance to select the most suitable pre-treatments and algorithms for Origin discrimination of whole green coffee beans. Three groups of different geographical origins were discriminated to reveal the distinctiveness of Yemeni coffee using databases where spectra were categorized as Group1: Yemen vs. Other origins (n = 884), Group 2: Yemen vs. Africa, Asia, Central America, and South America (n = 880), and Group 3: Yemeni Regions (Al Mahwit, Dhamar, Ibb, Sa'dah, and Sana'a (n = 496)). Principal Component Analysis (PCA) on the mean-centered matrix (900–2400 nm) was obtained using full random cross-validation and algorithm Nonlinear estimation by Iterative Partial Least Square (NIPALS) as a first step to observe spectral features, score trends, and identify outliers and dominant peaks in the loadings (Unscrambler® X v.10.4 software, CAMO Analytics, Oslo, Norway). Two- and three-dimensional figures were created using Microsoft Excel (Microsoft® Office Professional Plus™ 2021, Microsoft Corporation®) and JMP® 17.0 (SAS Institute Inc., Cary, NC, USA).

## Linear discriminant analysis

Based on the Bayes formula, linear discriminant analysis (LDA) identifies comparable spectral features for intra-class groups and differential spectral features to distinguish the classes or, in this case, the geographical origin of whole green coffee beans^49,50^. Before applying LDA for spectra classification, the dimensionality of each spectral database is reduced using PCA. The PCA-LDA combines the simplicity of PCA with the discriminant power of LDA^51^. Compared to other algorithms, such as Soft Independent Modeling of Class Analogy (SIMCA), PCA-LDA may have a reduced risk of overfitting, especially when dealing with a limited number of samples per class. It provides a linear combination of variables that maximally separates different classes in the reduced-dimensional space. The discriminant axes in PCA-LDA have a clear interpretation, as they are derived from the original variables. This can aid in understanding the features that contribute most to class separation^51^. Three spectra datasets were generated using Microsoft Excel (Microsoft® Office Professional Plus™ 2021, Microsoft Corporation®) for each discrimination group to test for mathematical pre-processing and modeling bias in the discriminant analysis; first, the Test set was organized by randomly selecting samples and all of their replicated spectra into a separate file. Next, the Training and Cross-validation sets were established using stratified random sampling sorting and an equal number of NIR spectra from the remaining samples for each geographical origin into an 80/20% distribution. This process ensured the homogeneity of variance and weight of the datasets by correcting for the disproportion and diversity of the total number of samples and spectra (Table 1). By utilizing a Top-Down strategy for PC selection methodology^52,53^, ten PCA-LDA models (900–2400 nm) were tested within each group utilizing PCs (principal components), which explained between 95% and 99.9% of the variation of the Training datasets using the Mahalanobis method (Unscrambler® X v.10.4 software, CAMO Analytics, Oslo, Norway).

In order to evaluate the effectiveness of the classification method, values from the Confusion Matrix were reported to describe the percentage of accuracy, sensitivity, and specificity (Microsoft Excel, Microsoft® Office Professional Plus™ 2021, Microsoft Corporation®). The percentage of correctly categorized spectra out of all the spectra in the datasets is measured by the accuracy. The sensitivity test described by Eq. (1) quantifies the discriminant model's ability to correctly identify the true origin of the green coffee beans spectra where TP = True positive and FN = False negative^38,43,54^. For the discrimination Group 1: Yemen vs. Other origins, the correctly classified spectra from Yemen were accounted as the TP values. For groups 2 and 3, the values from the Confusion Matrix were calculated for each origin and designated following the guidelines for Multi-Class Classification^55^, where TP = the only correctly classified spectra for each origin, FN = the incorrectly classified spectra on the row of each origin.

1 $$Sensitivity\;\% = \left( {\frac{{TP}}{{TP + FN}}} \right) \times 100$$

The specificity of the PCA-LDA model is the ability of the model to identify the true negatives correctly, which is represented by Eq. (2), where TN = True negative and FP = False positive^38,43,54^. For Group 1 the correctly classified spectra from Other origins were accounted as the TN values. For Groups 2 and 3, TN = all the other columns and rows from the Confusion Matrix where origins are considered the class "b" as the reference focus, FP = the incorrectly classified spectra on the column of each origin^55^.

2 $$Specificity\; \%=\left(\frac{TN}{TN+FP}\right)\times100$$

The prediction equations resulting from the Training set were applied to the Cross-validation and Test sets described in Table 1 and evaluated with the same quality parameters of accuracy, sensitivity, and specificity.

## Results

### Green coffee beans spectral profile

**Figure 1:**
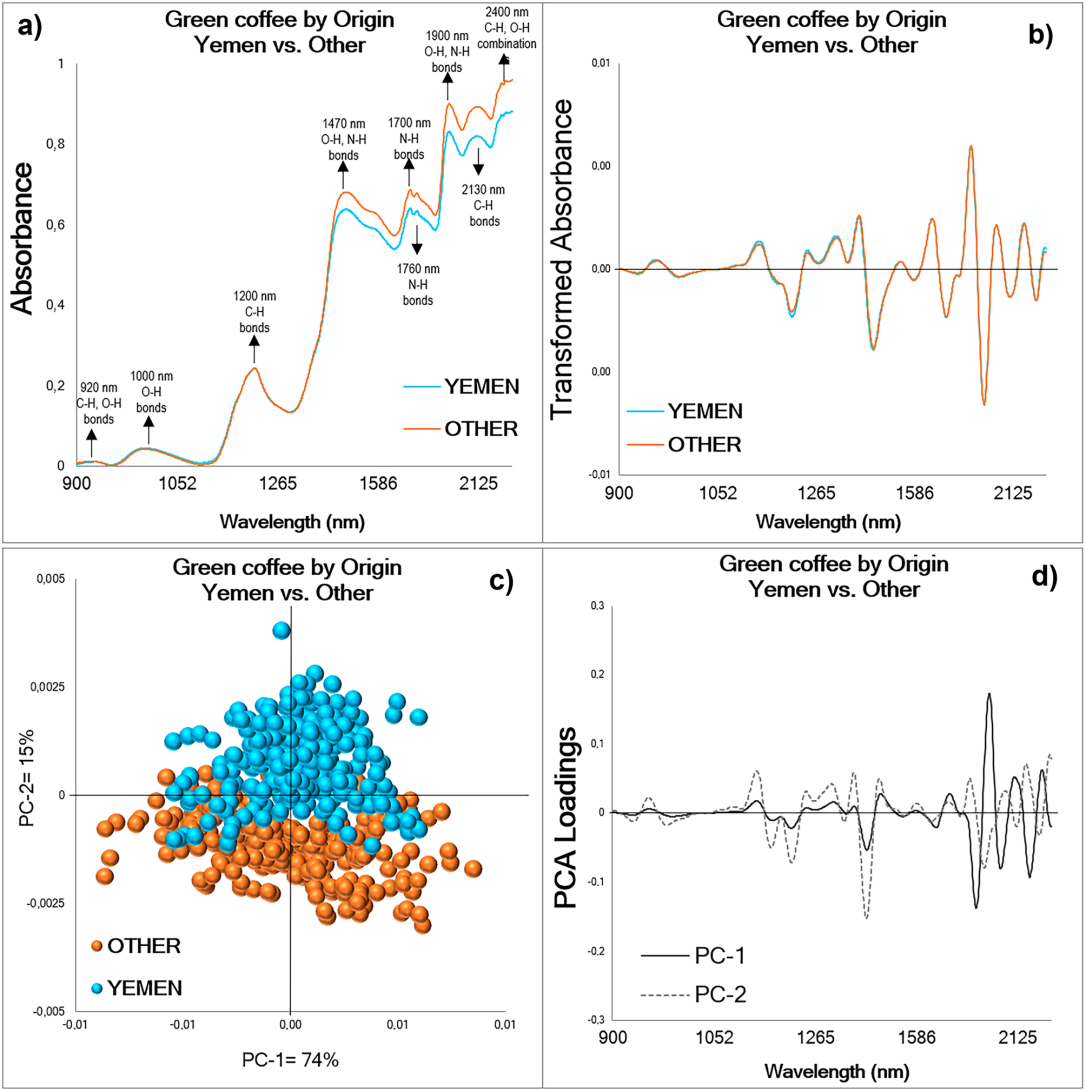
Exploratory analysis of whole green coffee beans NIR spectra (900–2400 nm) acquired from Group 1: Yemen and Other origins (n = 884). (**a**) Raw averaged NIR spectral signatures with Baseline Correction by geographical origin show the characteristic green coffee spectral pattern. (**b**) Transformed or processed averaged spectra showed no difference between origins. (**c**) PCA scores plot for samples from both origins containing the scores from the first two PCs explaining 89% of the total variance. (**d**) PCA loadings displaying the dominant peaks influencing the trends in the scores plot: PC-1 = 74%, PC-2 = 15%.

In the observed mean raw NIR spectra with Baseline Correction of whole green coffee beans from Yemen and Other origins (Fig. 1a), distinct peaks in absorbance were identified at 920, 1000, 1200, 1470, 1700, 1760, 1900, 2130, and 2400 nm^41,56^. These peaks are attributed to specific vibrational modes of chemical bonds in the coffee samples. The absorbance peaks at 920, 1000, and 1200 nm can be primarily assigned to O–H stretching vibrations, while the peaks at 1470, 1700, and 1760 nm correspond to C–H stretching vibrations. Notably, the absorbance peaks in the range of 1900 to 2400 nm are associated with a combination of O–H, C–H, and N–H vibrations, indicating the presence of multiple chemical components^33,50^. Interestingly, from 1400 to 2400 nm, there is an evident difference in the absorbance pattern between the spectra for green coffee from Yemen compared to Other origins, where the latest showed higher absorbances. In this region, the concentration of the main chemical compounds of green coffee, such as caffeine (C_8_H_10_N_4_O_2_), trigonelline (C_7_H_7_NO_2_), chlorogenic acid (C_16_H_18_O_9_), proteins, amino acids (RCH(NH_2_)COOH), lipids (CH_3_(CH_2_)_n_COOH), carbohydrates (C_x_(H_2_O)_y_), sucrose (C_12_H_22_O_11_), and water (H_2_O) have been reported to be correlated with the NIR spectrum^31,32,36^. Further, using a second derivative and Savitzky-Golay smoothing in the mathematical pre-treatment (Fig. 1b) enhances the visibility of these vibrational modes^57,58^. However, in this instance, no discernible disparities between the analyzed geographical origins were seen, emphasizing the necessity of carrying out more supervised pattern recognition techniques to unveil the real differences and demonstrate the technique's potential in discriminating coffee samples from different geographical origins.

The exploratory analysis carried out using PCA showed chemical similarities in the transformed spectra for whole green coffee beans from Yemen and Other origins between 900 and 2400 nm, where the scores of both geographical origins overlap. In the two-dimensional PCA scores plot (Fig. 1c), the first two Principal Components (PCs) explained 89% of the variation of the entire spectral database (n = 884). When labeling the quadrants from the scores plot as quadrant I, II, III, and IV, starting with the upper right corner as quadrant I and then turning left to label the others, it can be observed that the majority of scores for the Yemeni coffee samples can be seen in quadrants I and II. By contrast, most of the scores for samples from Other origins can be observed in quadrants III and IV. The Hotelling's T^2^ influence plot (not shown) did not reveal any outliers, ruling out the potential of artificial bias. The PC loadings provided insights into the dominant peaks influencing the tendencies observed in the scores plot (Fig. 1d). Specifically, PC-1 (926, 955, 984, 1130, 1158, 1214, 1240, 1310, 1367, 1410, 1455, 1660, 1728, 1868, 1934, 2020, 2093, 2224, 2310 nm) and PC-2 (929, 946, 1129, 1164, 1185, 1207, 1240, 1304, 1376, 1407, 1448, 1541, 1588, 1655, 1720, 1780, 1832, 1905, 2039, 2115, 2181, 2285 nm) explained 74% and 15% of the variance of the spectral database, respectively. An in-depth examination of the PC loadings reveals the specific vibrational modes associated with these dominant peaks. The PC-1, which accounts for the majority of the variance, is primarily associated with O–H stretching vibrations (e.g., 926, 955, 984 nm), C–H stretching vibrations (e.g., 1130, 1158, 1240 nm), and N–H vibrations (e.g., 1367, 1410 nm). Significant peaks in PC-1 can also be attributed to combinations of stretching and bending modes involving these functional groups. On the other hand, PC-2, contributing to a smaller portion of the variance, exhibits peaks corresponding to a combination of O–H, C–H, and N–H vibrations, involving both stretching and bending modes (e.g., 1129, 1164, 1207, 1240 nm)^33,50^.

## Geographical origin discrimination

**Figure 2:**
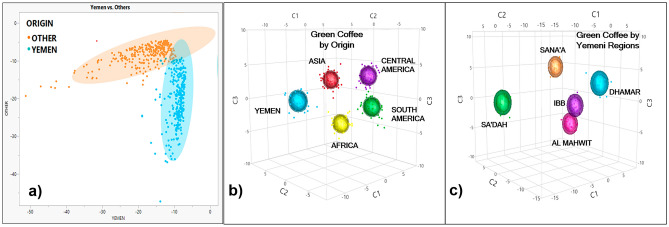
Training dataset trends in the PCA-LDA plots for whole green coffee beans NIR spectra (900–2400 nm) discrimination by geographical origin. (**a**) Group 1: Yemen vs. Other origins. (**b**) Group 2: Yemen vs. Africa, Asia, Central America, and South America. (**c**) Group 3: Yemeni regions (Al Mahwit, Dhamar, Ibb, Sa'dah, and Sana'a).

Table 2 contains the findings from the PCA-LDA performed on the three balanced databases created for each of the three discrimination groups; the results with the number of PCs or factors with the best performance can be seen in the table; the remaining results from the Top-Down approach for PCs selection are included in the Supplementary Information Online (Table S2, S4, S5). When comparing an equal number of NIR spectra of green coffee beans from Group 1: Yemen vs. Other origins, the same trends and patterns as previously described in Fig. 1 are obtained. In this case, six PCs, explaining 99.1 ± 0.01 of the variance, exhibit the best performance for spectra classification; the results from other PCs can be found in Supplementary Table S2 online. On average, the Training set of this supervised pattern recognition approach gave accuracy, sensitivity, and specificity of 98.5 ± 0.1, 96.9 ± 0.2, and 100 ± 0.0%, respectively. These values indicate that only 3.1 ± 0.3% of the green coffee beans spectra from Other origins were classified as false negatives, and none of the spectra for green coffee beans from Yemen corresponded to false positive values. All the Training databases displayed similar trends in the PCA-LDA plot where two defined groups are observed; in Fig. 2a, one of the obtained plots is shown as a representation of the three databases analyzed. The derived prediction equations from the Training were then applied to the Cross-validation and Test sets of spectra (Table 2). In the Cross-validation, average values of 98.8 ± 0.9, 97.6 ± 1.8, and 100 ± 0.0% for the accuracy, sensitivity, and specificity, respectively, were obtained. Additionally, the Test set containing the spectra from the excluded samples during the Training and Cross-validation processes was accurately classified with an average percentage of 95.9 ± 1.5%, a sensitivity of 94.6 ± 1.8%, and a specificity of 100 ± 0.0% when applying the prediction model. Consistent findings align with prior research where the geographical origin of green coffee was successfully distinguished through the application of NIRS (Supplementary Table S3 online).

**Table 2:** Results for the PCA-LDA spectra classification and model quality parameters for balanced databases or interactions from whole green coffee bean samples from different geographical origins.

RIGIN				PCA-LDA Mahalanobis (900–2400 nm)					
				Training		Cross-validation		Test	
G	PCs	%Explained Variance	Parameter	Correctly classified	Total	Correctly classified	Total	Correctly classified	Total
1	6	99.1 ± 0.01	YEMEN	273 ± 0.5	282	68 ± 1.2	70	137 ± 2.6	144
			OTHER	282 ± 0.0	282	70 ± 0.0	70	36 ± 0.0	36
			Ac (%)	**98.5 ± 0.1**		**98.8 ± 0.9**		**95.9 ± 1.5**	
			Se (%)	**96.9 ± 0.2**		**97.6 ± 1.8**		**94.6 ± 1.8**	
			Sp (%)	**100 ± 0.0**		**100 ± 0.0**		**100 ± 0.0**	
2	9	99.7 ± 0.01	YEMEN	48 ± 0.0	48	12 ± 0.0	12	432 ± 18.1	436
			AFRICA	45 ± 1.4	48	10 ± 1.7	12	27 ± 6.2	40
			ASIA	41 ± 0.9	48	10 ± 1.7	12	39 ± 5.1	68
			C_AMERICA	44 ± 0.9	48	11 ± 0.0	12	6.0 ± 4.3	12
			S_AMERICA	47 ± 0.8	48	12 ± 0.0	12	20 ± 1.7	24
			Ac (%)	**93.5 ± 1.7**		**92.8 ± 5.7**		**70.5 ± 14.0**	
			Se (%)	**94.0 ± 1.6**		**95.6 ± 2.8**		**74.0 ± 13.2**	
			Sp (%)	**96.8 ± 0.7**		**95.6 ± 2.5**		**92.7 ± 3.1**	
3	7	99.6 ± 0.04	AL MAHWIT	19 ± 1.7	22	4 ± 0.5	6	2.0 ± 1.2	4
			DHAMAR	22 ± 0.0	22	6 ± 0.0	6	19 ± 4.1	28
			IBB	22 ± 0.0	22	6 ± 0.5	6	80 ± 13.4	108
			SA'DAH	21 ± 0.9	22	6 ± 0.0	6	3.0 ± 2.2	8
			SANA'A	22 ± 0.5	22	6 ± 0.5	6	102 ± 21.5	208
			Ac (%)	**96.7 ± 2.8**		**92.2 ± 4.7**		**57.6 ± 19.1**	
			Se (%)	**99.1 ± 1.3**		**97.8 ± 3.1**		**51.3 ± 15.9**	
			Sp (%)	**98.4 ± 1.1**		**96.2 ± 1.1**		**78.0 ± 2.2**	

G: Group; PCs: Principal Components; Ac: Accuracy; Se: Sensitivity; Sp: Specificity. C: Central; S: South.

Average ± standard deviation values are in bold.

**Figure 3:**
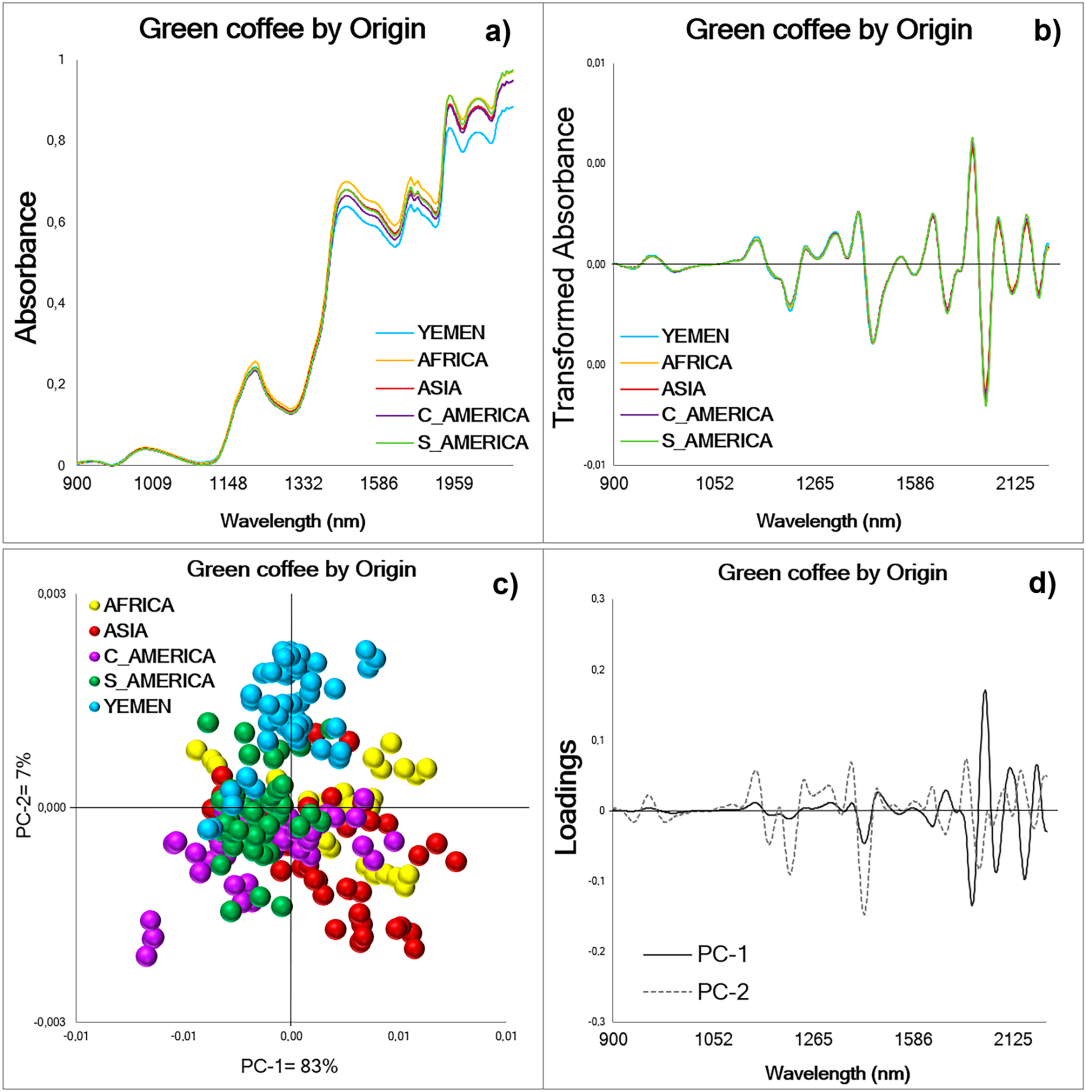
Whole green coffee beans NIR spectra (900–2400 nm) acquired from Group 2: Yemen, Africa, Asia, Central America, and South America (n = 240). (**a**) Raw averaged NIR spectral signatures using balanced databases with Baseline Correction by geographical origin. (**b**) Transformed or processed averaged spectra by geographical origin using balanced databases. (**c**) PCA scores plot for an equal number of samples from all origins, the first two PCs explaining 90% of the total variance. (**d**) PCA loadings displaying the dominant peaks influencing the trends in the scores plot: PC-1 = 83%, PC-2 = 7%. C: Central; S: South.

The same trends and patterns as those shown in Fig. 1 are observed for Group 2 when comparing an equivalent number of NIR spectra for green coffee beans from Yemen with those from Africa, Asia, Central America, and South America (Fig. 3). The averaged raw absorbance with Baseline Correction displayed higher values for the green coffee beans from Africa, followed by South America, Asia, Central America and lastly Yemen in the wavelength range between 1400–2400 nm (Fig. 3a). In the averaged transformed absorbance, no differences can be observed between origins (Fig. 3b). In the two-dimensional PCA scores plot the first two PCs explained 90% of the variance of the database (n = 240) and trends can be observed by geographical origin, however, they overlap (Fig. 3c). The dominant peaks in the PC loadings influencing the trends in the scores plot explained 83% and 7% of the variance of the spectral database, respectively (Fig. 3d). In the PCA-LDA for Group 2, nine PCs explaining 99.7 ± 0.01 of the variance, exhibit the best performance (Table 2), the results from other PCs can be found in Supplementary Table S4 online. During the Training process of the PCA-LDA (Fig. 2b) an accuracy, sensitivity, and specificity of 93.5 ± 1.7, 94.0 ± 1.6, and 96.8 ± 0.7%, respectively, were obtained. These values indicate that 6.0 ± 1.7% of the green coffee bean spectra from Yemen, Africa, Asia, Central America, and South America were classified as false negatives, and 3.2 ± 0.7% of the green coffee spectra corresponded to false positive values. The Cross-validation revealed average values of 92.8 ± 5.7, 95.6 ± 2.8, and 95.6 ± 2.5% for the accuracy, sensitivity, and specificity, respectively. When using the prediction model, the spectra in the Test set were correctly classified with an average percentage of 70.5 ± 14%, a sensitivity of 74.0 ± 13.2%, and a specificity of 92.7 ± 3.1% (Table 2).

**Figure 4:**
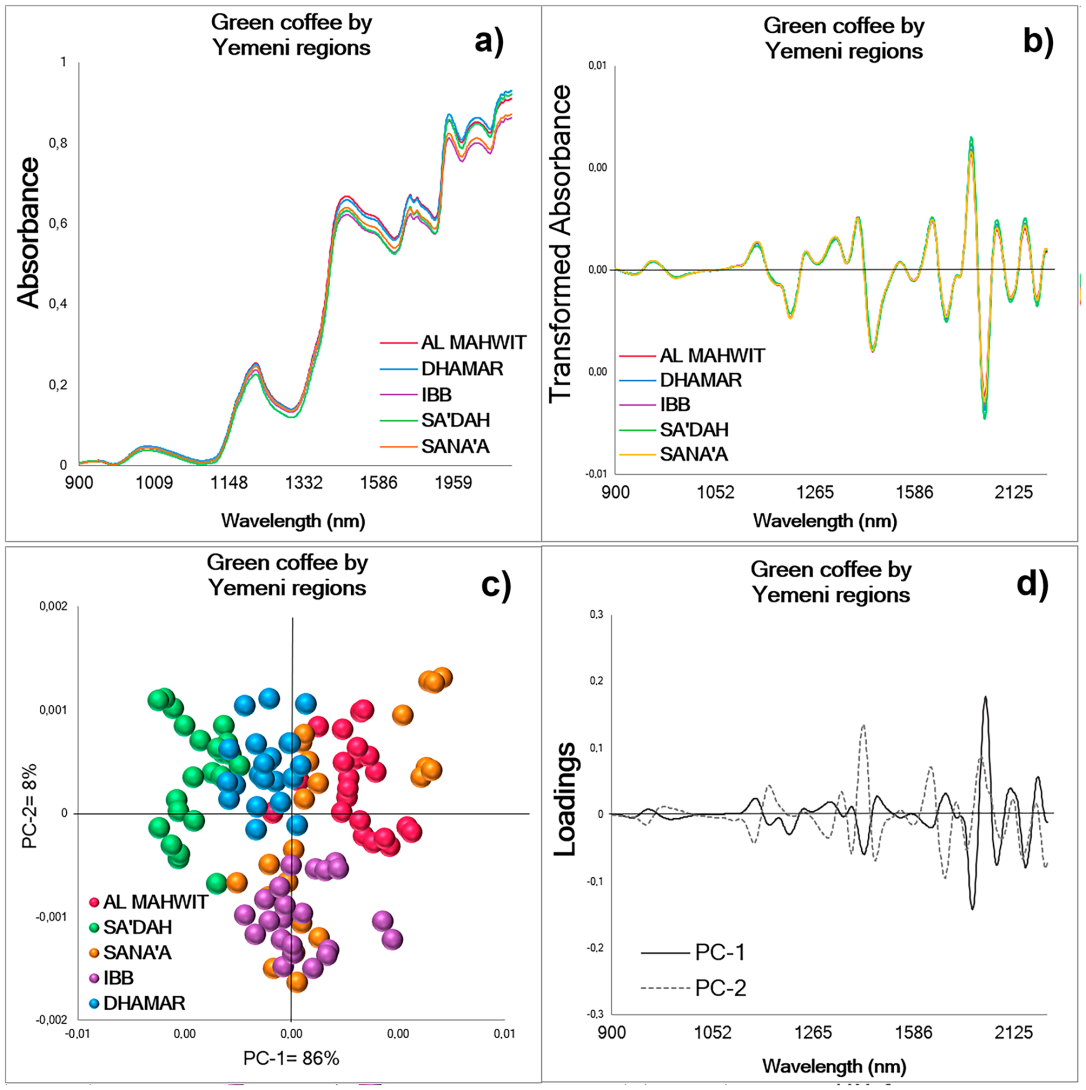
Whole green coffee beans NIR spectra (900–2400 nm) acquired from Group 3: Yemeni regions; Al Mahwit, Dhamar, Ibb, Sa'dah, and Sana'a (n = 110). (**a**) Raw averaged NIR spectral signatures with Baseline Correction by region using balanced databases. (**b**) Transformed or processed averaged spectra by region using balanced databases. (**c**) PCA scores plot for an equal number of samples from all regions, the first two PCs explaining 94% of the total variance. (**d**) PCA loadings displaying the dominant peaks influencing the trends in the scores plot: PC-1 = 86%, PC-2 = 8%.

The averaged NIR spectra of green coffee beans from Group 3: Yemeni regions exhibited differences in the wavelength range between 900 and 2400 nm, where samples from Al Mahwit and Dhamar showed the highest absorbance values followed by Sa'dah, Sana'a, and Ibb (Fig. 4a). In the averaged transformed absorbance, some differences can be observed in the wavelength range between 2000 and 2400 nm (Fig. 4b) with Sa'dah showing the highest absorbance. In the two-dimensional PCA scores plot the first two PCs explained 94% of the variance of the database (n = 110) and trends with almost no overlapping can be observed by region for Al Mahwit, Dhamar, Ibb, and Sa'dah; the scores for samples from Sana'a are the only ones without a defined trend (Fig. 4c). The dominant peaks in the PC loadings influencing the trends in the scores plot explained 86% and 8% of the variance of the spectral database, respectively (Fig. 4d). In the PCA-LDA for Group 3, seven PCs explaining 99.6 ± 0.04 of the variance, exhibit the best performance (Table 2), the results from other PCs can be found in Supplementary Table S5 online. The PCA-LDA training process for Yemeni regions (Fig. 2c) revealed an accuracy, sensitivity, and specificity of 96.7 ± 2.8, 99.1 ± 1.3, and 98.4 ± 1.1%, respectively. These values are almost 10% higher than the ones obtained in the general PCA-LDA using all the samples from the Yemeni regions (Supplementary Table S6 online), demonstrating the importance of using balanced databases (Table 2) and indicating that 0.9 ± 1.3% of the green coffee beans spectra were classified as false negatives, and only 1.6 ± 1.1% of the green coffee spectra corresponded to false positives values. The Cross-validation revealed average values of 92.2 ± 4.7, 97.8 ± 3.1, and 96.2 ± 1.1% for the accuracy, sensitivity, and specificity, respectively. In the Test set, spectra were correctly classified with an average percentage of 57.6 ± 19.1%, a sensitivity of 51.3 ± 15.9%, and a specificity of 78.5 ± 2.2% (Table 2), revealing the need to add more samples in the Training set to generate a more global and robust model that could predict the Yemeni region from new samples.

## Discussion

In the field of NIRS discrimination concerning the origins of green coffee (Supplementary Table S3 online), this investigation distinguishes itself as a thorough exploration spanning multiple coffee-producing countries (Table 2, Fig. 2), thereby presenting a holistic aspect that significantly contributes to understanding the complexities characterizing the green coffee profile. Despite the role of Yemeni coffee in the history of coffee and its unique attributes, quality, and "terroir," there has been very limited scientific work to explore the chemical distinctiveness of Yemeni coffee in comparison to other origins^11–13^. Some studies which delved into the genetic makeup of Yemeni coffee plants have unveiled a tapestry of diversity that sets Yemen apart from all other coffee-growing nations^7,8,14–16^. This study, incorporating over 200 samples from major coffee regions worldwide, is a pioneering endeavor to elucidate the discriminative capabilities of NIR spectra of whole green coffee beans from Yemen and other regions, including Africa, Asia, Central America, and South America. The emphasis on Yemeni coffee fulfills a dual objective: differentiating coffees within Yemen's own regions and distinguishing Yemeni coffee from those of other geographic origins. Moreover, given Yemeni coffee's role as a socioeconomic lifeline and the prevalent fraud affecting its exports, this research underscores the significance of developing traceability and sustainability tools specifically for Yemeni coffee.

Analytical methods for origin verification of food products such as honey, wine, olive oil, tea, spices, dairy, and meat have been developed since the 1980s and have proven to be very efficient^59,60^. For over a decade, NIRS and isotope fingerprinting have been considered potential methods for geographical origin discrimination in coffee^37,61^. Isotope fingerprinting presents some advantages when compared with NIRS. Isotope ratios, particularly those of elements such as oxygen and carbon, provide unrivaled specificity to the geographical origin of coffee beans, revealing the unique climatic conditions under which they develop^20^. This approach is less affected by processing differences, resulting in consistent findings even with roasted or ground samples. The long-term stability of isotope ratios allows for retrospective study, useful for historical research or investigating long-term stability^62^. In this context, isotope fingerprinting of green Yemeni coffee has been previously described using inductively coupled plasma-optical emission spectroscopy (ICP-OES)^29^. In that study, 16 green coffee samples collected from eight major coffee-producing Yemeni regions: Al Hudaydah, Al Mahwit, Dhamar, Hajjah, Lahij, Raymah, Sana'a, and Taiz were completely distinguished from Ethiopian coffee by Ca- and Na-content and PCA that explained 100% of the variation^29^. However, NIRS has significant advantages over isotopic fingerprinting. NIRS is a non-destructive, fast technology that requires little sample preparation, making it ideal for large-scale and routine analysis^33,50^. Its capacity to extract a wide range of chemical information from the complete sample enables a more thorough characterization of coffee, taking into account numerous components such as caffeine, chlorogenic acids, proteins, carbohydrates, and lipids^31^. After calibration, NIRS also gives real-time results, allowing for swift supply chain decisions^20^. In contrast to isotope fingerprinting, NIRS does not require the use of hazardous materials or costly laboratory setups, making it more accessible and cost-effective for both research and industrial applications^33^.

In the present study, the variation in NIR spectral signatures (Figs. 1a, 3a, 4a), coupled with the successful differentiation of green Yemeni coffee beans in comparison to other geographical origins (Table 2, Fig. 2), confirms the different chemical composition of the beans associated with the "terroir" of where the coffees were cultivated. The NIR spectral signature, which is a combination of different chemical measures, is capable of capturing a wide range of changes, including those impacted by genetic diversity, post-harvest processing techniques, and environmental factors, in addition to origin-specific features^63–66^. Consequently, the NIR spectral signature undergoes alterations, providing a plausible explanation for the varying absorbances detected in the raw NIR spectra with Baseline Correction between Yemeni green coffee and counterparts from different origins and within distinct Yemeni regions^33,50^. While the chemical composition introduces variation, PCA-LDA retains its effectiveness in discerning the geographical origin of coffee samples (Table 2, Fig. 2)^37,67^. This study emphasizes the robustness of these methods, grounded in the capacity of NIR spectral signatures to encapsulate a diverse array of variations, solidifying its pivotal role as a powerful tool in the traceability of coffee origin. In addition, the present study represents a significant advancement over the existing research landscape in the discrimination of the geographical origin of green coffee. While prior studies have demonstrated the efficacy of NIRS and chemometrics in this context (Supplementary Table S3 online)^37–44^, the specific case of Yemeni coffee has been addressed in only one prior study utilizing the SIMCA algorithm^40^. There, the NIR spectra (1200–1500 nm) from commercially available whole green coffee samples purchased in Japanese markets and from Cuba (n = 70), Ethiopia (n = 76), Indonesia (n = 178), Tanzania (n = 51), and Yemen (n = 70) were subjected to discrimination with accuracies of 73%, 93%, 82%, 80%, and 100%, respectively^40^. It is important to note that Yemeni coffee is subject to specific discrimination, which is similar to the findings of the current study. However, the present investigation surpasses limitations in several key aspects. Firstly, the study incorporates a more extensive dataset, encompassing over 120 samples from various Yemeni regions, ensuring a more comprehensive and representative analysis of the discriminative potential of NIR spectra in distinguishing Yemeni coffee from other origins. Secondly, unlike the previous study that focused on commercially available samples from Japanese markets, the present research deliberately includes whole green coffee beans obtained directly from smallholder coffee farms in Yemen, adding a very strong level of authentication for the samples' origins. Thirdly, the application of PCA-LDA algorithm, providing a robust analytical framework that merits discussion in comparison to the SIMCA algorithm. Therefore, the present manuscript not only expands the scope and depth of research in this field but also contributes novel insights and methodological advancements, establishing it as a noteworthy contribution to the scientific understanding of coffee origin discrimination.

Future applications on NIRS and chemometrics for origin discrimination of green coffee beans must account for the enrichment of the database by the expansion of the geographical coverage, including more possible regional and global origins, the addition of chemical analysis, more post-harvest processes, diverse cultivation practices, and genetic varieties according to coffee markets and potential user needs and the introduction of coffee from different species, such as *C. canephora* to allow an iterative improvement of the authentication power, enhance the robustness of the findings and to overcome the limitations of the PCA-LDA^51^. By identifying the geographical origin of whole green coffee beans using NIRS and chemometrics, Yemeni smallholder coffee farmers can gain recognition for their distinctive products, receive premium prices for their coffee, rebuild the reputation of Yemeni coffee in domestic and international markets, and ultimately protect and conserve Yemeni coffee cultivation and heritage. Similarly, buyers could use this cost-effective and fast technology to confirm coffee origin information and protect themselves against fraudulent coffees. The utilization of such a technology can work to improve the economic sustainability of Yemeni farmers and Yemen's economy, as well as contribute to strengthening traceability and transparency protocols in the wider coffee industry.

## Conclusion

This study elucidates the potential use of NIRS coupled with chemometrics, particularly the discriminant analysis (PCA-LDA), as a robust tool for ensuring the traceability of green coffee beans from various origins (Yemen, Africa, Asia, Central America, and South America). Through a comprehensive consideration of the information encapsulated in the NIR spectra of whole green coffee beans, chemometric models were meticulously developed. These models exhibited a remarkable capability to accurately predict the geographical origin of Yemeni coffee samples against Other origins, achieving an accuracy, sensitivity, and specificity surpassing 98% when utilizing balanced databases. The precision of these prediction models establishes a foundation for creating fast and cost-effective authentication tools, effectively safeguarding against fraud, empowering farmers, and providing buyers with enhanced insights into the quality and distinctive characteristics of the respective coffee beans and bolstering the sustainability of the Yemeni coffee sector. Subsequent research endeavors should focus on refining the existing models, encompassing a thorough evaluation of the chemical composition of Yemeni green coffee. Additionally, the enrichment of the database with samples from diverse regions, harvest seasons, genetic varieties, chemical analysis, and processing methods will contribute to the development of a more robust, reliable, and efficient prediction model.

### Supplementary Information

Supplementary Tables.

## Data Availability

Data is available upon request from Dr. Mariana Santos-Rivera (Mariana@Smartspectra.ai).

## References

[1] Sepúlveda WS, Chekmam L, Maza MT, Mancilla NO (2016). Consumers’ preference for the origin and quality attributes associated with production of specialty coffees: Results from a cross-cultural study. Food Res. Int..

[2] Lordemann, J. A., Mora, C. & Mulder, N. The main drivers of arabica coffee prices in Latin America. in *Economic Commission for Latin America and the Caribbean (ECLAC)* (2021).

[3] Williams SD, Barkla BJ, Rose TJ, Liu L (2022). Does coffee have terroir and how should it be assessed?. Foods.

[4] Barbosa Escobar F, Petit O, Velasco C (2021). Virtual terroir and the premium coffee experience. Front. Psychol..

[5] Muñoz-Pajares AJ, Várzea V, Silva M, do C.  (2023). The story of coffee: Legend and truth. Trends Plant Sci..

[6] Al-Najjar, A., Dijkxhoorn, Y., Zubiry, R. & Ruben, R. *Understanding coffee farming practices and prospects in Yemen : case study from Bani Matar*. https://research.wur.nl/en/publications/829554cb-d9b5-4e9b-977a-f9227a064ae5 (2023) 10.18174/589422.

[7] Montagnon C, Mahyoub A, Solano W, Sheibani F (2021). Unveiling a unique genetic diversity of cultivated *Coffea arabica* L. in its main domestication center: Yemen. Genet. Resour. Crop Evol..

[8] Montagnon C, Sheibani F, Benti T, Daniel D, Bote AD (2022). Deciphering early movements and domestication of coffea arabica through a comprehensive genetic diversity study covering Ethiopia and Yemen. Agronomy.

[9] Obadi SM (2017). Competitive advantage of Yemeni export in the US market. Open Access Libr. J..

[10] Greeney, A. Yemen’s Traditional and Resilient Coffee Sector: Production Totals Steady from 1690 to Present. (Harvard University Division of Continuing Education, 2022).

[11] Omer, A. E.-E. Determination of engineering and chemical properties of some Yemeni coffee varieties. in *Agricultural mechanization and engineering Between existing and prospected* 497–512 (2008).

[12] Nogaim QA, Al-Duais M, Al-Warafi A, Al-Erianee H, Al-Sayadi M (2013). The chemical composition of Yemeni green coffee. J. Food Chem. Nutr..

[13] Ahmed Ali AM (2022). Evaluation of the chemical constituents, antioxidant and enzyme inhibitory activities of six Yemeni green coffee beans varieties. Food Biosci..

[14] Al-Murish TM, Elshafei AA, Al-Doss AA, Barakat MN (2013). Genetic diversity of coffee (*Coffea arabica* L.) in Yemen via SRAP, TRAP and SSR markers. J. Food Agric. Environ..

[15] Hussein MAA, Al-Azab AAA, Habib SS, Sherif FME, El-Garhy HAS (2017). Genetic diversity, structure, and DNA fingerprint for developing molecular ids of Yemeni coffee (*Coffea arabica* L.) germplasm assessed by SSR markers. Egypt J. Plant Breed.

[16] Silvestrini M (2007). Genetic diversity and structure of Ethiopian, Yemen and Brazilian *Coffea arabica* L. accessions using microsatellites markers. Genet. Resour. Crop Evol..

[17] Al-sabai A, Neszmélyi GI (2019). The challenges and actual questions of the agriculture in Yemen. Stud. Mundi Econ..

[18] Mohamed, H., Elayah, M. & Schuplen, L. Yemen between the Impact of the Climate Change and the Ongoing Saudi-Yemen War: A Real Tragedy. (2017).

[19] International Trade Centre (ITC). *Niche Markets for Coffee: Specialty, Environment and Social Aspects*. https://intracen.org/fr/media/12305 (2012).

[20] Perez M, Domínguez-López I, López-Yerena A, Vallverdú Queralt A (2023). Current strategies to guarantee the authenticity of coffee. Crit. Rev. Food Sci. Nutr..

[21] Thorburn Burns D, Tweed L, Walker MJ (2017). Ground roast coffee: review of analytical strategies to estimate geographic origin, species authenticity and adulteration by dilution. Food Anal. Methods.

[22] Ferreira T, Galluzzi L, De Paulis T, Farah A (2021). Three centuries on the science of coffee authenticity control. Food Res. Int..

[23] *The coffee exporter’s guide*. (International Trade Centre, 2011).

[24] Breisinger, C., Raouf, M. & Wiebelt, M. *Prioritizing agricultural value chains for reviving the food system in Yemen: Input for an agricultural strategy update*. https://ebrary.ifpri.org/digital/collection/p15738coll2/id/133552 (2020) 10.2499/p15738coll2.133552.

[25] Muharram I, Alsharjabi KM (2019). Sustainable agriculture, food security and the role of agricultural research and technology transfer in Yemen. Syr. J. Agric. Res..

[26] Sanchez, C., Boot, W., Roche, D. & Ilyas, M. *Rediscovering coffee in Yemen, updating the coffee value chain and a marketing strategy to re-position Yemen in the international coffee markets*. https://static1.squarespace.com/static/6006ff76e391125d24308c5c/t/605cd65445ca547a26f1a2ca/1616696927496/1.+USAID+Final-Report_Rediscovering-Coffee-in-Yemen_August-2013.pdf (2013).

[27] Chen Y, Gao B, Lu W (2023). Recent research advancements of coffee quality detection: Targeted analyses vs. nontargeted fingerprinting and related issues. J. Food Qual..

[28] Pruvot-Woehl S (2020). Authentication of *Coffea arabica* varieties through DNA fingerprinting and its significance for the coffee sector. J. AOAC Int..

[29] Mohammed F, Guillaume D, Dowman S, Abdulwali N (2019). An easy way to discriminate Yemeni against Ethiopian coffee. Microchem. J..

[30] Sigma Aurum F, Imaizumi T, Manasikan T, Praseptiangga D, Nakano K (2022). Coffee origin determination based on analytical and nondestructive approaches –a systematic literature review. Rev. Agric. Sci..

[31] Munyendo L, Njoroge D, Hitzmann B (2021). The potential of spectroscopic techniques in coffee analysis—a review. Processes.

[32] Barbin DF, Felicio ALDSM, Sun DW, Nixdorf SL, Hirooka EY (2014). Application of infrared spectral techniques on quality and compositional attributes of coffee: An overview. Food Res. Int..

[33] Williams P, Antoniszyn J, Manley M (2019). Near Infrared Technology: Getting the best out of light.

[34] Belchior V, Botelho BG, Casal S, Oliveira LS, Franca AS (2020). FTIR and chemometrics as effective tools in predicting the quality of specialty coffees. Food Anal. Methods.

[35] Belchior V, Botelho BG, Franca AS (2022). Comparison of spectroscopy-based methods and chemometrics to confirm classification of specialty coffees. Foods.

[36] Belchior V, Franca AS, Oliveira LS (2016). Potential of diffuse reflectance infrared fourier transform spectroscopy and chemometrics for coffee quality evaluation. ETP Int. J. Food Eng..

[37] Villegas AM (2014). Identificación de origen y calibración para tres compuestos químicos en café, por espectroscopia de infrarojo cercano. Cenicafe.

[38] Marquetti I (2016). Partial least square with discriminant analysis and near infrared spectroscopy for evaluation of geographic and genotypic origin of arabica coffee. Comput. Electron. Agric..

[39] Bona E (2017). Support vector machines in tandem with infrared spectroscopy for geographical classification of green arabica coffee. LWT - Food Sci. Technol..

[40] Okubo N, Kurata Y (2019). Nondestructive classification analysis of green coffee beans by using near-infrared spectroscopy. Foods.

[41] Giraudo A (2019). Determination of the geographical origin of green coffee beans using NIR spectroscopy and multivariate data analysis. Food Control.

[42] Wongsaipun S (2021). Application of artificial neural network for tracing the geographical origins of coffee bean in Northern areas of thailand using near infrared spectroscopy. Chiang Mai J. Sci..

[43] Nguyen Minh Q (2022). Authenticity green coffee bean species and geographical origin using near-infrared spectroscopy combined with chemometrics. Int. J. Food Sci. Technol..

[44] Dharmawan A, Masithoh RE, Amanah HZ (2023). Development of PCA-MLP model based on visible and shortwave near infrared spectroscopy for authenticating Arabica coffee origins. Foods.

[45] Montagnon C, Rossi V, Guercio C, Sheibani F (2022). Vernacular names and genetics of cultivated coffee (*Coffea arabica*) in Yemen. Agronomy.

[46] Fernández-Cabanás VM, Garrido-Varo A, Pérez-Marín D, Dardenne P (2006). Evaluation of pretreatment strategies for near-infrared spectroscopy calibration development of unground and ground compound feedingstuffs. Appl. Spectrosc..

[47] Liu Y (2019). The influence of spectral pretreatment on the selection of representative calibration samples for soil organic matter estimation using vis-NIR reflectance spectroscopy. Remote Sens..

[48] Huang J, Romero-Torres S, Mojgan M (2010). Practical considerations in data pre-treatment for NIR and Raman spectroscopy. Am. Pharm. Rev..

[49] Fearn T (2009). Regression statistics in discriminant analysis. NIR News.

[50] Pasquini C (2003). Near infrared spectroscopy: Fundamentals, practical aspects and analytical applications. J. Braz. Chem. Soc..

[51] Lasalvia M, Capozzi V, Perna G (2022). A comparison of PCA-LDA and PLS-DA techniques for classification of vibrational spectra. Appl. Sci..

[52] Messick NJ, Kalivas JH, Lang PM (1997). Selecting factors for partial least squares. Microchem. J..

[53] Barros AS, Rutledge DN (1998). Genetic algorithm applied to the selection of principal components. Chemom. Intell. Lab. Syst..

[54] Parikh R, Mathai A, Parikh S, Sekhar GC, Thomas R (2008). Understanding and using sensitivity, specificity and predictive values. Indian J. Ophthalmol..

[55] Grandini, M., Bagli, E. & Visani, G. Metrics for Multi-Class Classification: an Overview. Preprint at http://arxiv.org/abs/2008.05756 (2020).

[56] Adnan A, von Hörsten D, Pawelzik E, Mörlein D (2017). Rapid prediction of moisture content in intact green coffee beans using near infrared spectroscopy. Foods.

[57] Ostertagová E, Ostertag O (2016). Methodology and application of Savitzky-Golay moving average polynomial smoother. Glob. J. Pure Appl. Math..

[58] Fearn T (2016). Look at the (Pre-Treated) spectra. NIR News.

[59] Liu H (2023). A review of recent compound-specific isotope analysis studies applied to food authentication. Food Chem..

[60] Nobari Moghaddam H, Tamiji Z, Akbari Lakeh M, Khoshayand MR, Haji Mahmoodi M (2022). Multivariate analysis of food fraud: A review of NIR based instruments in tandem with chemometrics. J. Food Compos. Anal..

[61] Rodrigues CI (2009). Stable isotope analysis for green coffee bean: A possible method for geographic origin discrimination. J. Food Compos. Anal..

[62] Sim J, Mcgoverin C, Oey I, Frew R, Kebede B (2023). Stable isotope and trace element analyses with non-linear machine-learning data analysis improved coffee origin classification and marker selection. J. Sci. Food Agric..

[63] Tsegay G (2020). Effect of altitude of coffee plants on the composition of fatty acids of green coffee beans. BMC Chem..

[64] Getachew M (2022). The relationship between elevation, soil temperatures, soil chemical characteristics, and green coffee bean quality and biochemistry in southwest Ethiopia. Agron. Sustain. Dev..

[65] Worku M, de Meulenaer B, Duchateau L, Boeckx P (2018). Effect of altitude on biochemical composition and quality of green arabica coffee beans can be affected by shade and postharvest processing method. Food Res. Int..

[66] Cheng B, Furtado A, Smyth HE, Henry RJ (2016). Influence of genotype and environment on coffee quality. Trends Food Sci. Technol..

[67] Posada H, Ferrand M, Davrieux F, Lashermes P, Bertrand B (2009). Stability across environments of the coffee variety near infrared spectral signature. Heredity.

