# Supplementary material for: Identifying the origin of Yemeni green coffee beans using near infrared spectroscopy: a promising tool for traceability and sustainability
Source: Sci Rep. 2024 Jun 10;14:13342. doi: 10.1038/s41598-024-64074-9 (PMC11164903; doi:10.1038/s41598-024-64074-9)
Supplement: Supplementary file 1 — Supplementary Tables. [file 41598_2024_64074_MOESM1_ESM.pdf]

Identifying the Origin of Yemeni Green Coffee Beans Using Near Infrared Spectroscopy: A Promising Tool for Traceability and Sustainability.

Mariana Santos-Rivera[1*], Christophe Montagnon[2], and Faris Sheibani[1,3]

[1] Smartspectra Limited, 52b Fitzroy Street, London W1T 5BT, UK
[2] RD2 Vision, 60 rue du Carignan, 34270 Valflaunes, France
[3] Qima Coffee, 21 Warren Street, Fitzrovia, London W1T 5LT, UK

*Corresponding author: Mariana Santos-Rivera; Email: Mariana@Smartspectra.ai
ORCID: https://orcid.org/0000-0002-3356-6276

Supplementary Table S1. Number of specialty green coffee samples by geographical origin (n=221) contributing to the NIR spectra databases (n=884).

GROUP	ORIGIN	Samples	NIR Spectra
**YEMEN**	AL MAHWIT	8	32
	DHAMAR	14	56
	IBB	34	136
	SAADA	9	36
	SANA'A	59	236
	**Total**	**124**	**496**
**AFRICA**	BURUNDI	2	8
	ETHIOPIA	10	40
	KENYA	10	40
	TANZANIA	1	4
	UGANDA	2	8
	**Total**	**25**	**100**
**ASIA**	CHINA	9	36
	INDIA	11	44
	INDONESIA	5	20
	MYANMAR	3	12
	TIMOR	4	16
	**Total**	**32**	**128**
**CENTRAL AMERICA**	GUATEMALA	3	12
	HONDURAS	3	12
	MEXICO	6	24
	PANAMA	4	16
	SALVADOR	2	8
	**Total**	**18**	**72**
**SOUTH AMERICA**	BOLIVIA	3	12
	BRAZIL	5	20

GROUP	ORIGIN	Samples	NIR Spectra
	COLOMBIA	11	44
	PERU	2	8
	**Total**	**21**	**84**
**OCEANIA**	PAPUA	1	4
**Total**		**221**	**884**

Supplementary Table S2. PCA-LDA results in average ± standard deviation from the Top-Down selection approach for PCs selection for Group 1: Yemen vs. Other origins. PCs: Principal Components; Ac: Accuracy; Se: Sensitivity; Sp: Specificity.

#PCs	% Explained variance	Parameter	PCA-LDA Mahalanobis 900-2400 nm		
			Training	Cross-validation	Test
3	93.6 ± 0.19	Ac (%)	91.5 ± 0.4	94.0 ± 0.9	89.4 ± 1.7
		Se (%)	87.7 ± 0.4	91.0 ± 1.8	89.6 ± 2.0
		Sp (%)	95.3 ± 0.9	97.1 ± 0.8	90.7 ± 9.4
4	97.3 ± 0.08	Ac (%)	95.7 ± 0.8	97.1 ± 1.0	93.5 ± 2.6
		Se (%)	93.0 ± 0.9	95.2 ± 1.3	93.3 ± 4.2
		Sp (%)	98.5 ± 0.6	99.0 ± 0.7	94.4 ± 7.9
5	98.3 ± 0.01	Ac (%)	96.6 ± 0.3	97.4 ± 1.2	94.1 ± 1.8
		Se (%)	94.3 ± 0.5	97.1 ± 1.2	92.6 ± 2.3
		Sp (%)	98.9 ± 0.3	97.6 ± 3.4	100 ± 0.0
**6**	**99.1 ± 0.01**	**Ac (%)**	**98.5 ± 0.1**	**98.8 ± 0.9**	**95.9 ± 1.5**
		**Se (%)**	**96.9 ± 0.2**	**97.6 ± 1.8**	**94.6 ± 1.8**
		**Sp (%)**	**100 ± 0.0**	**100 ± 0.0**	**100 ± 0.0**
7	99.4 ± 0.01	Ac (%)	99.2 ± 0.3	99.8 ± 0.3	98.7 ± 1.1
		Se (%)	98.8 ± 0.6	100 ± 0.0	98.4 ± 1.4
		Sp (%)	99.5 ± 0.2	99.5 ± 0.7	100 ± 0.0
8	99.5 ± 0.01	Ac (%)	99.3 ± 0.1	99.8 ± 0.3	98.7 ± 1.1
		Se (%)	98.9 ± 0.5	100 ± 0.0	98.6 ± 1.5
		Sp (%)	99.8 ± 0.3	99.5 ± 0.7	99.1 ± 1.3
9	99.6 ± 0.0	Ac (%)	99.6 ± 0.3	100 ± 0.0	98.9 ± 1.2
		Se (%)	99.2 ± 0.6	100 ± 0.0	99.1 ± 1.3
		Sp (%)	100 ± 0.0	100 ± 0.0	98.1 ± 1.3
10	99.7 ± 0.0	Ac (%)	99.6 ± 0.3	100 ± 0.0	99.1 ± 0.9
		Se (%)	99.2 ± 0.6	100 ± 0.0	99.1 ± 1.3
		Sp (%)	100 ± 0.0	100 ± 0.0	99.1 ± 1.3
11	99.8 ± 0.0	Ac (%)	99.6 ± 0.3	100 ± 0.0	99.1 ± 0.9
		Se (%)	99.2 ± 0.6	100 ± 0.0	99.1 ± 1.3
		Sp (%)	100 ± 0.0	100 ± 0.0	99.1 ± 1.3
12	99.8 ± 0.0	Ac (%)	99.6 ± 0.2	100 ± 0.0	99.3 ± 1.0
		Se (%)	99.2 ± 0.3	100 ± 0.0	99.1 ± 1.3
		Sp (%)	100 ± 0.0	100 ± 0.0	100 ± 0.0

Supplementary Table S3. Studies that reported the application of NIRS and chemometrics for the discrimination of the geographical origin of green coffee.

#	Year	n	Type	Origins	NIR region (nm)	Algorithm	Outcome
1	2014	55	Powder	Colombia: Caldas, Quindío, Antioquia, Cauca, Cesar.	1100-2500	PCA-LDA	Accuracy: 100% for Caldas, Quindío, and Antioquia, 91% for Cauca, and 95% for Cesar.[1]
2	2016	90	Powder	Brazil: Cornélio Procópio, Paranavaí, Mandaguari, Londrina.	1100-2500	PLS-DA	Accuracy: 100%.[2]
3	2017	74	Powder	Brazil:Cornélio Procópio, Paranavaí, Mandaguari, Londrina.	1100-2500	SVM	Accuracy: 100% for Paranavaí and Londrina; 99% for Cornélio Procópio and Mandaguari.[3]
4	2019	445	Beans	Cuba, Ethiopia, Indonesia, Tanzania, and Yemen.	1200-1500	SIMCA	Accuracies of: 86%, 73%, 93%, 80%, 100%, 80%, and 80%, respectively.[4]
5	2019	191	Beans	Brazil, Honduras, India, and Vietnam.	800-2800	PLS-DA	Accuracy: 97.6%, 99.5%, 97.5%, and 95.1% respectively.[5]
6	2021	59	Beans	Thailand: Chiang Mai, Lampang, and Mae Hong Son.	800-2500	ANN-SOMDI	Effectively distinguishing among the evaluated origins in clusters.[6]
7	2022	153	Beans	Vietnam: Dak Lak and non-Dak Lak	900-1700	PLS-DA	Accuracy: 93% for each origin.[7]
8	2023	NA	Beans	Indonesia: Temanggung, Toraja, Gayo, and Kintamani.	400-1630	ANN	Accuracy: 99.7%.[8]
9	2024	221	Beans	Yemen (Al Mahwit, Dhamar, Ibb, Sa'dah, and Sana'a), Africa, Asia, Central America, South America.	900-2400	PCA-LDA	Present Study. Accuracy: >98%.

Supplementary Table S4. PCA-LDA results in average ± standard deviation from the Top-Down selection approach for PCs selection for Group 2: Yemen vs. Africa, Asia, Central America, and South America. PCs: Principal Components; Ac: Accuracy; Se: Sensitivity; Sp: Specificity.

#PCs	% Explained variance	Parameter	PCA-LDA Mahalanobis 900-2400 nm		
			Training	Cross-validation	Test
3	94.7 ± 0.27	Ac (%)	56.9 ± 9.4	57.2 ± 12.0	50.2 ± 13.6
		Se (%)	54.2 ± 11.3	54.4 ± 10.3	43.2 ± 9.4
		Sp (%)	80.7 ± 3.0	80.9 ± 5.0	84.4 ± 3.5
4	97.8 ± 0.04	Ac (%)	70.7 ± 5.7	61.1 ± 14.5	55.6 ± 16.5
		Se (%)	68.0 ± 7.8	59.5 ± 16.3	48.6 ± 11.3
		Sp (%)	86.5 ± 2.6	82.5 ± 4.5	87.1 ± 2.2
5	98.7 ± 0.01	Ac (%)	72.4 ± 5.9	66.7 ± 10.5	58.9 ± 12.6
		Se (%)	69.1 ± 8.3	63.8 ± 14.9	54.9 ± 10.7
		Sp (%)	87.2 ± 2.5	84.7 ± 5.8	88.3 ± 2.8
6	99.2 ± 0.03	Ac (%)	80.7 ± 4.5	75.6 ± 9.3	56.7 ± 12.5
		Se (%)	80.5 ± 6.3	78.1 ± 10.2	56.9 ± 12.6
		Sp (%)	90.9 ± 1.5	88.5 ± 4.4	89.4 ± 2.8
7	99.5 ± 0.00	Ac (%)	87.4 ± 2.9	83.9 ± 7.6	64.7 ± 10.7
		Se (%)	87.9 ± 3.8	84.8 ± 7.1	67.4 ± 10.7
		Sp (%)	93.9 ± 1.0	92.3 ± 2.3	90.8 ± 3.2
8	99.6 ± 0.02	Ac (%)	91.9 ± 2.4	85.6 ± 6.3	65.2 ± 12.3
		Se (%)	92.9 ± 2.3	87.0 ± 6.0	68.3 ± 11.2
		Sp (%)	96.1 ± 0.9	93.1 ± 1.9	91.3 ± 2.9
**9**	**99.7 ± 0.01**	**Ac (%)**	**93.5 ± 1.7**	**92.8 ± 5.7**	**70.5 ± 14.0**
		**Se (%)**	**94.0 ± 1.6**	**95.6 ± 2.8**	**74.0 ± 13.2**
		**Sp (%)**	**96.8 ± 0.7**	**95.6 ± 2.5**	**92.7 ± 3.1**
10	99.8 ± 0.01	Ac (%)	96.4 ± 1.3	92.8 ± 8.3	69.1 ± 11.1
		Se (%)	96.7 ± 1.3	93.3 ± 7.3	72.1 ± 9.9
		Sp (%)	98.2 ± 0.4	96.5 ± 2.6	93.8 ± 1.8
11	99.8 ± 0.01	Ac (%)	97.9 ± 1.1	95.6 ± 5.3	72.0 ± 10.3
		Se (%)	98.5 ± 0.9	97.2 ± 2.9	76.3 ± 9.0
		Sp (%)	99.0 ± 0.4	97.8 ± 2.0	94.0 ± 1.3
12	99.9 ± 0.01	Ac (%)	98.1 ± 1.4	96.1 ± 4.7	77.0 ± 8.7
		Se (%)	98.5 ± 1.1	97.8 ± 2.4	79.1 ± 7.3
		Sp (%)	99.0 ± 0.5	98.1 ± 2.2	94.7 ± 1.3

Supplementary Table S5. PCA-LDA results in average ± standard deviation from the Top-Down selection approach for PCs selection for Group 3: Yemeni Regions (Al Mahwit, Dhamar, Ibb, Sa'dah, and Sana'a). PCs: Principal Components; Ac: Accuracy; Se: Sensitivity; Sp: Specificity.

#PCs	% Explained variance	Parameter	PCA-LDA Mahalanobis 900-2400 nm		
			Training	Cross-validation	Test
3	95.8 ± 0.53	Ac (%)	75.2 ± 12.0	68.9 ± 22.9	41.5 ± 20.2
		Se (%)	78.8 ± 9.4	72.4 ± 20.8	36.2 ± 11.7
		Sp (%)	88.3 ± 3.5	85.6 ± 5.0	75.0 ± 4.2
4	97.9 ± 0.14	Ac (%)	86.4 ± 10.2	75.6 ± 21.0	49.8 ± 21.9
		Se (%)	92.1 ± 6.5	82.2 ± 18.3	44.1 ± 15.1
		Sp (%)	93.5 ± 3.7	88.4 ± 7.5	77.5 ± 4.1
5	98.8 ± 0.1	Ac (%)	93.3 ± 6.2	87.8 ± 11.0	57.9 ± 21.1
		Se (%)	98.2 ± 2.6	93.3 ± 9.4	51.4 ± 18.2
		Sp (%)	96.7 ± 2.4	94.1 ± 4.0	79.8 ± 3.0
6	99.4 ± 0.04	Ac (%)	95.8 ± 2.4	90.0 ± 7.4	57.7 ± 21.4
		Se (%)	99.1 ± 1.3	96.7 ± 4.7	52.0 ± 17.3
		Sp (%)	97.9 ± 1.0	95.2 ± 2.3	77.9 ± 2.8
**7**	**99.6 ± 0.04**	**Ac (%)**	**96.7 ± 2.8**	**92.2 ± 4.7**	**57.6 ± 19.1**
		**Se (%)**	**99.1 ± 1.3**	**97.8 ± 3.1**	**51.3 ± 15.9**
		**Sp (%)**	**98.4 ± 1.1**	**96.2 ± 1.1**	**78.0 ± 2.2**
8	99.7 ± 0.03	Ac (%)	98.5 ± 0.9	93.3 ± 3.1	55.6 ± 17.6
		Se (%)	100 ± 0.0	97.8 ± 1.6	47.8 ± 14.5
		Sp (%)	99.2 ± 0.4	96.8 ± 1.2	76.4 ± 1.5
9	99.8 ± 0.02	Ac (%)	98.8 ± 1.1	95.6 ± 3.1	57.4 ± 16.7
		Se (%)	100 ± 0.0	97.8 ± 1.6	49.7 ± 13.0
		Sp (%)	99.4 ± 0.6	97.8 ± 1.9	77.4 ± 2.0
10	99.8 ± 0.02	Ac (%)	99.7 ± 0.4	98.9 ± 1.6	60.2 ± 18.5
		Se (%)	100 ± 0.0	100 ± 0.0	47.9 ± 15.2
		Sp (%)	99.8 ± 0.2	99.4 ± 0.8	78.1 ± 5.1
11	99.9 ± 0.01	Ac (%)	99.7 ± 0.4	100 ± 0.0	56.3 ± 19.7
		Se (%)	100 ± 0.0	100 ± 0.0	48.8 ± 14.1
		Sp (%)	99.8 ± 0.2	100 ± 0.0	77.9 ± 2.9
12	99.9 ± 0.01	Ac (%)	100 ± 0.0	96.7 ± 3.1	59.2 ± 21.3
		Se (%)	100 ± 0.0	98.9 ± 1.6	47.3 ± 13.0
		Sp (%)	100 ± 0.0	98.4 ± 1.5	78.2 ± 3.6

Supplementary Table S6. General PCA-LDA spectra classification and quality parameters for whole green coffee beans from different geographical origins by three discrimination groups. PCs: Principal Components; Ac: Accuracy; Se: Sensitivity; Sp: Specificity.

GROUP	#PCs	Explained variance	ORIGIN	PCA-LDA Mahalanobis 900-2400 nm		
				Correctly classified	Incorrectly classified	Total
1	6	99.0	YEMEN	482	14	496
			OTHER	386	2	388
			TOTAL	868	16	884
			Ac (%)	**98.2**		
			Se (%)	**97.2**		
			Sp (%)	**99.5**		
2	9	99.6	YEMEN	496	0	496
			AFRICA	81	19	100
			ASIA	109	19	128
			CENTRAL AMERICA	49	23	72
			SOUTH AMERICA	81	3	84
			TOTAL	816	68	880
			Ac (%)	**92.7**		
			Se (%)	**88.9**		
			Sp (%)	**96.8**		
3	7	99.2	AL MAHWIT	5	27	32
			DHAMAR	53	3	56
			IBB	111	25	136
			SAADA	32	4	36
			SANA'A	227	9	236
			TOTAL	428	68	496
			Ac (%)	**86.3**		
			Se (%)	**92.3**		
			Sp (%)	**91.9**		

References

1. Villegas, A. M. *et al.* Identificación de origen y calibración para tres compuestos químicos en café, por espectroscopia de infrarojo cercano. *Cenicafe* **65**, 7–16 (2014).
2. Marquetti, I. *et al.* Partial least square with discriminant analysis and near infrared spectroscopy for evaluation of geographic and genotypic origin of arabica coffee. *Comput. Electron. Agric.* **121**, 313–319 (2016).
3. Bona, E. *et al.* Support vector machines in tandem with infrared spectroscopy for geographical classification of green arabica coffee. *LWT - Food Sci. Technol.* **76**, 330–336 (2017).
4. Okubo, N. & Kurata, Y. Nondestructive Classification Analysis of Green Coffee Beans by Using Near-Infrared Spectroscopy. *Foods* **8**, 82 (2019).
5. Giraudo, A. *et al.* Determination of the geographical origin of green coffee beans using NIR spectroscopy and multivariate data analysis. *Food Control* **99**, 137–145 (2019).
6. Wongsaipun, S. *et al.* Application of Artificial Neural Network for Tracing the Geographical Origins of Coffee Bean in Northern Areas of Thailand Using Near Infrared Spectroscopy. *Chiang Mai J. Sci.* **48**, 163–175 (2021).
7. Nguyen Minh, Q. *et al.* Authenticity green coffee bean species and geographical origin using near-infrared spectroscopy combined with chemometrics. *Int. J. Food Sci. Technol.* **57**, 4507–4517 (2022).
8. Dharmawan, A., Masithoh, R. E. & Amanah, H. Z. Development of PCA-MLP Model Based on Visible and Shortwave Near Infrared Spectroscopy for Authenticating Arabica Coffee Origins. *Foods* **12**, 2112 (2023).
